# Transcriptomic study of Herpes simplex virus type-1 using full-length sequencing techniques

**DOI:** 10.1038/sdata.2018.266

**Published:** 2018-11-27

**Authors:** Zsolt Boldogkői, Attila Szűcs, Zsolt Balázs, Donald Sharon, Michael Snyder, Dóra Tombácz

**Affiliations:** 1 Department of Medical Biology, Faculty of Medicine, University of Szeged, Szeged, 6720, Hungary; 2 Department of Genetics, School of Medicine, Stanford University, Stanford, California, 94305, USA

**Keywords:** Gene expression, RNA sequencing, Herpes virus, Transcriptomics

## Abstract

Herpes simplex virus type-1 (HSV-1) is a human pathogenic member of the *Alphaherpesvirinae* subfamily of herpesviruses. The HSV-1 genome is a large double-stranded DNA specifying about 85 protein coding genes. The latest surveys have demonstrated that the HSV-1 transcriptome is much more complex than it had been thought before. Here, we provide a long-read sequencing dataset, which was generated by using the RSII and Sequel systems from Pacific Biosciences (PacBio), as well as MinION sequencing system from Oxford Nanopore Technologies (ONT). This dataset contains 39,096 reads of inserts (ROIs) mapped to the HSV-1 genome (X14112) in RSII sequencing, while Sequel sequencing yielded 77,851 ROIs. The MinION cDNA sequencing altogether resulted in 158,653 reads, while the direct RNA-seq produced 16,516 reads. This dataset can be utilized for the identification of novel HSV RNAs and transcripts isoforms, as well as for the comparison of the quality and length of the sequencing reads derived from the currently available long-read sequencing platforms. The various library preparation approaches can also be compared with each other.

## Background & Summary

Herpes simplex virus type-1 (HSV-1) is a human pathogenic herpesvirus belonging to the family of *Herpesviridae*. HSV-1 is associated with orofacial infections^1^. This is a highly contagious and lifelong infection that is widespread in the world^2^. HSV-1 is a prototype herpesvirus, which is used as a model organism in the study of molecular pathogenesis of the lytic and latent viral infection, and it is also used as a gene delivery vector^3,4^ for basic research and gene therapy^5^, as well as in oncolytic virotherapy^6^. HSV-1 has a large (~152 kbp) double-stranded DNA genome and a very complex transcriptome^7^.

**Figure 1:**
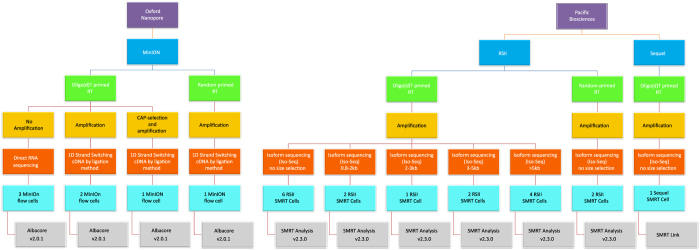
Data flow diagram shows the detailed overview of this research.

Here, we provide a large RNA-seq dataset derived from third- and fourth-generation^8^ long-read sequencing (LRS) methods (Fig. 1). The previously applied short-read sequencing approaches are inefficient in distinguishing between embedded gene products, RNA isoforms and overlapping transcripts. This problem can be circumvented by LRS methods that are capable of identifying full-length transcripts^9–13^. Our major aim with this study was to provide a dataset that can be suitable for deeply characterizing the complexity of the HSV-1 transcriptome profile and for obtaining a detailed picture on the transcript-level variations. In order to achieve this goal, the currently available LRS techniques, the Pacific Biosciences (PacBio) and the Oxford Nanopore Technologies (ONT) platforms were used for the characterisation of the HSV-1 lytic transcriptome. We used the RSII^7^ and the Sequel isoform sequencing (Iso-Seq) approaches for the analyses of both the polyadenylated fraction and the total transcriptome of the HSV-1 transcripts. The amplified Iso-Seq method was used for cDNA sequencing. We used the ONT MinION technique for both cDNA and direct (d)RNA sequencing for the investigations of the poly(A) RNA molecules.

The utilized sequencing methods along with the various library preparation approaches were as follows, PacBio: oligo(d)T-primed and random-hexamer primed Iso-Seq protocol with or without size-selection, as well as ONT: cDNA-sequencing, cDNA-sequencing using 5′ Cap-selected transcripts, and dRNA sequencing. These techniques allow for the detection of novel transcript isoforms (splice and length variants), as well as several novel protein-coding RNAs, non-coding (nc)RNAs, and polycistronic transcripts. This LRS dataset is also useful for the investigation of the transcriptional overlaps. Additionally, these data can be used to compare the “sequencing by synthesis” method with the nanopore-based approach. The various library preparation approaches can also be compared with each other.

We used 17 SMRT Cells for the sequencing of our samples on RSII system, using the P6-C4 chemistry. The movie time was 240 or 360 per SMRT Cell. Samples were also run on a single Sequel SMRT Cell using P6-C4 chemistry with 600 min movie time. Altogether 7 MinION flow cells were applied for sequencing the different ONT libraries. The raw reads were aligned to the HSV-1 reference genome in each case (X14112).

**Figure 2:**
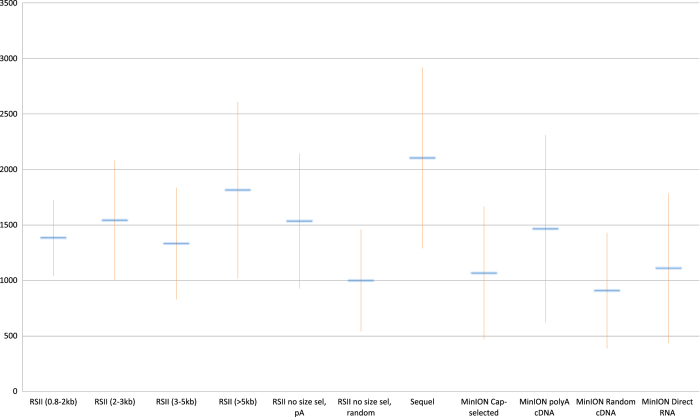
The average read lengths in the various library preparation and sequencing methods. Error bars represents the standard deviance (SD).

The PacBio RSII sequencing generated 38,601 reads of inserts (ROIs) using oligo(d)T-primed reverse transcription (RT), while, using the Sequel platform, we obtained 77,851 HSV-1 specific ROIs. Overall, the different ONT methods generated 175,169 viral reads (Table 1). The average read lengths are as follows: 1,536 and 1,000 bp for the no size-selected oligo(d)T-primed and random-primed RSII, respectively; 2,104 for the Sequel, while the obtained read lengths are 1,466, 1,067 and 1,111 bp for the MinION cDNA, Cap-selected cDNA, and direct RNA-seq methods (Table 2, Fig. 2).

The dataset generated here is expected to provide valuable information for the herpesvirus studies and will open new possibilities for the investigation of HSV-1 at the molecular level. Our data provides a wide-ranging resource for understanding, comparing, and analysing the utilized library preparation and sequencing methods, as well as this valuable data can be used to design novel bioinformatics pipelines and also to test the currently available ones.

**Figure 3:**
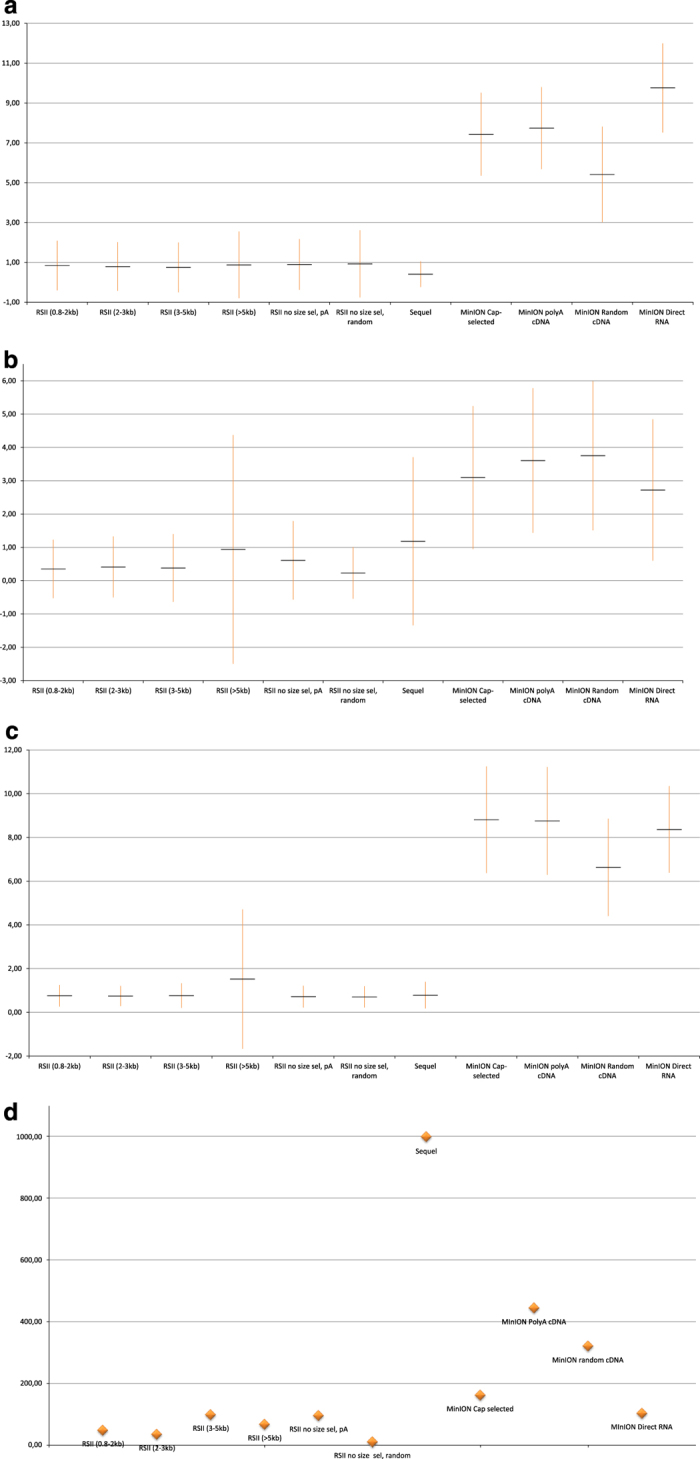
Summary of the read qualities gained from the utilized long-read sequencing approaches. (**a**) Percentage of deletions; error bar represents the SD values. (**b**) Percentage of insertions with SD values. (**c**) Percentage of mismatches given together with SD values. (**d**) Average read coverages across the HSV-1 genome.

**Figure 4:**
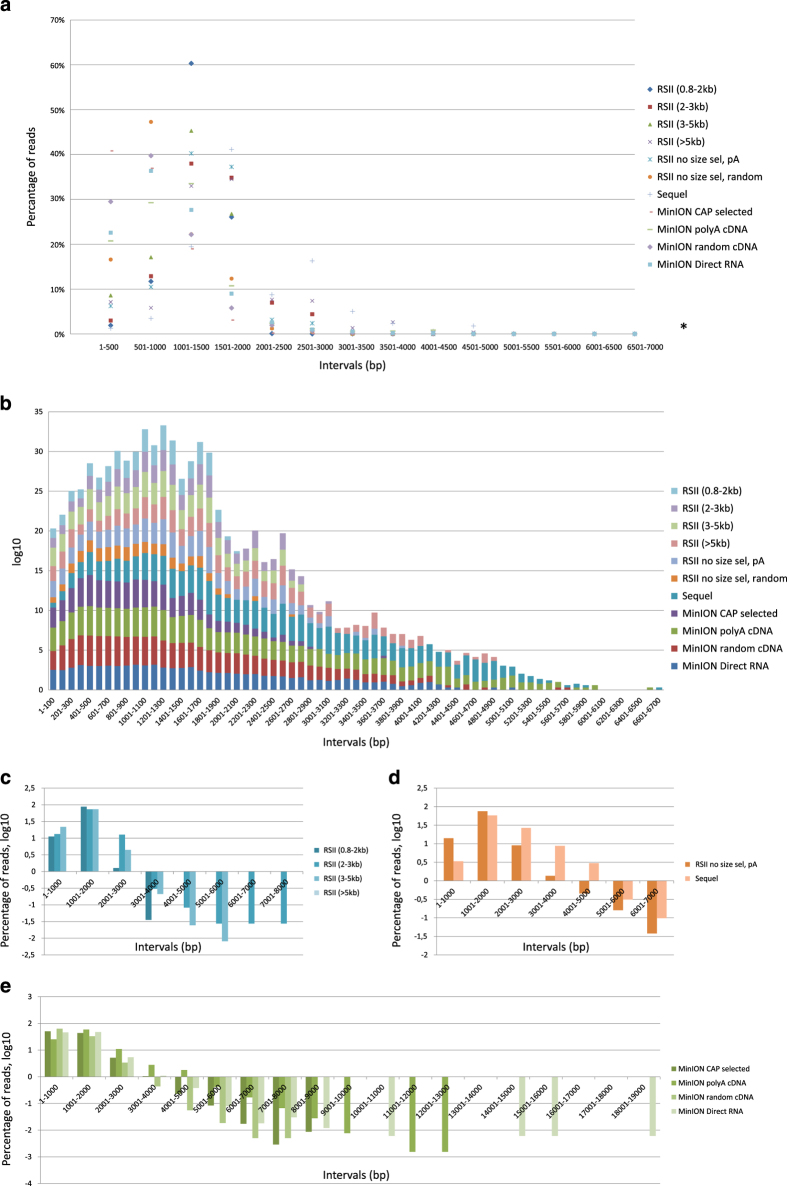
Read length distributions. (**a**) The average distribution of read lengths which align to the HSV-1 genome; binned to 500 bp intervals. *Percentage values are in Figure 3. (**b**) The divided bar chart shows the number of reads at log10 scale, binned to 100 bp intervals. The samples can be seen broken down according to the library preparation and/or sequencing approaches in C–E. (**c**) Read lengths distributions at log10 scale, binned to 1,000 bp ranges. This figure shows the RSII size-selected samples. It can be seen that the majority of the reads mapped to the HSV-1 genome are shorter than 3,000 bps, most of the reads are between 1,001–2,000 bps, independently from the focus of the size selection. (**d**) This bar chart shows that the same library preparation method (Isoform sequencing with no size selection using Clontech SMARTer PCR cDNA Synthesis Kit) result in different read length distribution using the two PacBio platforms: 40% of the Sequel reads are longer than 2,000 bp, while only 10% of the RSII reads belong to this size range. (**e**) The average read length using the MinION nanopore sequencing is below 2,000 bp. There is no random primed read above 9,000 bp. Intriguingly, the longest reads are derived from the direct RNA sequencing approach (15,000–19,000 bp).

**Figure 5:**
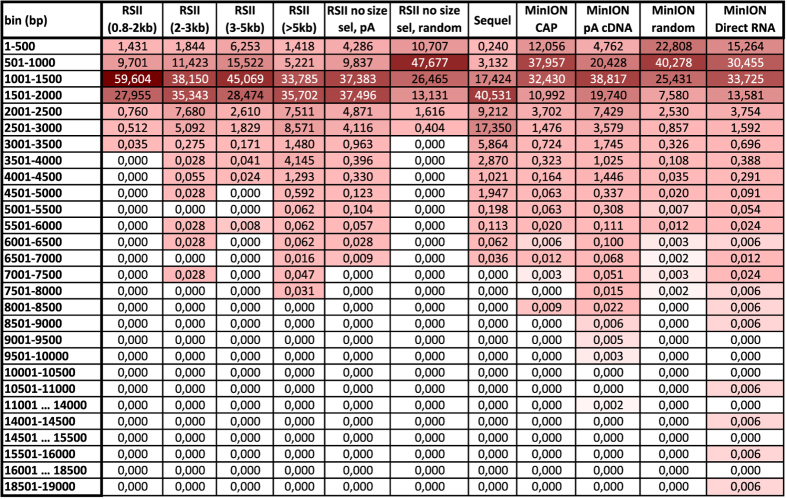
Percentage values of number of read counts binned to 500 bp. This heat map-like illustration shows the distribution of read counts within 500 bp intervals. This dataset shows that the Sequel sequencer resulted in longer average read lengths (~79% of the reads are longer than 1,500 bp). The size selection of the RSII samples had a significant effect on the average read lengths: even if most of the reads are within 1,000–2,000 bp, the distribution of the read counts is different: 0.8–2 kb: 87,560%; 2–3 kb: 73,493%; 3–5 kb: 73,543%; >5 kb: 69,487% of the reads are within this range. The MinION sequencing on the poly(A)-selected samples shows about the same read length distribution as the non-size-selected RSII (the majority of the reads is within the 1,000–2,000 bp interval, however, the distribution of the MinION reads is smoother. The Cap-selected samples and the direct RNA samples have a peak at the lower range: about 30–35% of the reads are between 501–1,000 and 1,001–1,500 bp in both samples. The random primed samples (RSII and MinION) generated the shortest average read lengths.

**Figure 6:**
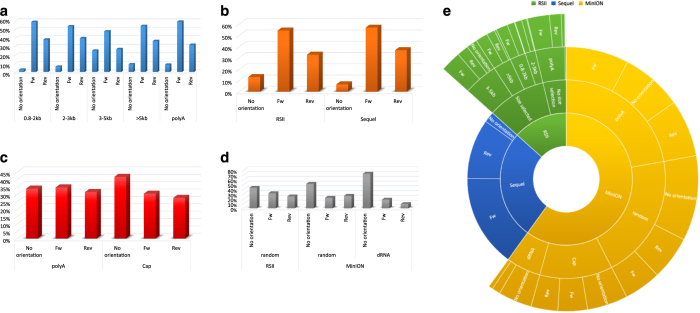
Comparing the Porechop results derived from the analysis of different libraries. (**a**) This figure shows the percentages of read counts derived from different PacBio RSII poly(A) sequencing. (**b**) This bar chart compares the data derived from the two PacBio approaches. We could detect the adapters on more than 90% Sequel reads, while we obtained in a poorer result from RSII. (**c**) This diagram compares the ONT’s own library preparation approach (1D) with the Lexogen Teloprime Cap-selection protocol, which used for a library, run on a MinION flow cell. (**d**) We were not able to identify the orientation of about 50% of reads using the random-primed approach, while dRNA-seq could detect adapters on about 20% of the reads. (**e**) Segmented pie chart illustrates the distribution of reads with (labelled as: Fw and Rev) or without orientation (labelled as: No orientation). Fifty-six % of this dataset derived from the different nanopore sequencing approaches, 26.5% from Sequel and the remaining less than 20% is from the RSII. A high ratio (~90%) of reads from PacBio RSII and Sequel sequencing could be categorized as Fw or Rev, however Porechop were not able to identify the adapters and thus the orientation of at least about 30% of reads.

Here, we provide a comprehensive overview about preparation methods of sequencing libraries, as well as a description of the data (Figure 1, Table 3). Detailed statistics about the read quality, such as insertions, deletions, and mismatches, as well as the coverages of the pre-processed data (binary alignment (BAM)) can be found in Table 4 and Fig. 3. The data show that the ONT MinION sequencing resulted in relatively high error rates for insertions, deletions and mismatches. The best read quality (i.e. less insertions, deletions and mismatch) – as expected – was obtained from the PacBio runs. The composition of the errors of the three sequencers (RSII, Sequel, MinION) are different. Mismatches are the most common errors in ONT, while the insertions are the least frequent errors in ONT (consistent with the previously published data^14^). Intriguingly, contrary to others’ datasets^14^, deletions are the major errors in our RSII data; the Sequel shows the same pattern as was expected^14^. The read length distribution of the samples is visualized in Fig. 4 and Fig. 5 Porechop tool (https://github.com/rrwick/Porechop) was used to carry out an analysis on the 5′ and 3′ adapters, with which we were able to determine the orientation of the sequencing reads (Table 5 (available online only), Table 6, Fig. 6). About 90% of the PacBio reads could be categorized as forward or reverse oriented read, but only 66%, 58% and 49% from the 1D-seq, Cap-seq and random libraries could be categorized, respectively.

**Figure 7:**
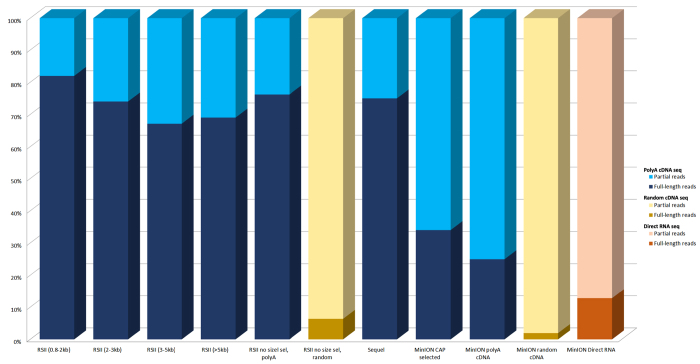
Bar chart represents the full-length and partial read distributions in the different libraries.

The ratio of the complete (full-length) reads in the different samples were also calculated (Fig. 7). The data shows that the random-primed cDNA sequencing as well as the dRNA sequencing resulted in very few complete reads, which has a technical reason: random primed sequencing could gain complete reads when the random primer binds to the 3′-end of the RNA. The small number of full-length reads, from the dRNA sequencing is consistent with our previous findings: we observed that the dRNA sequencing resulted in poor 5′ and 3′ read ends^15,16^. It can also be seen that nanopore sequencing produces a large number of incomplete reads; these short fragments are probably removed from PacBio sequencing by the MagBead loading.

## Methods

A part of the methods section (PacBio RSII sequencing) is an expanded version of descriptions in our related work^7^, however, the largest part of the data including the entire PacBio Sequel and ONT sequencing data has not yet been published elsewhere.

The various library preparation and sequencing methods utilized in this study are shown in Figure 1.

### Viruses, cells and infection

The Vero immortalized kidney epithelial cell line, derived from the kidney of an African green monkey (*Cercopithecus aethiops*) was used for maintaining and propagating HSV-1. The cell culture was grown in Dulbecco’s modified Eagle medium (DMEM, Gibco/Thermo Fisher Scientific) with 10% Foetal Bovine Serum (Gibco/Thermo Fisher Scientific) and 100 μl/ml Penicillin-Streptomycin 10 K/10 K Mixture (Lonza), and in a 37 °C incubator with a humidified atmosphere of 5% CO_2_ in air. The viral stocks were prepared by infecting rapidly-growing semi-confluent cells with viruses at a multiplicity of infection (MOI) of 1 plaque-forming unit (pfu)/cell. The infected cells were incubated until complete cytopathic effect was observed. Three times freeze-thaw cycles were applied and it was followed by a centrifugation step at 10,000×g for 15 min. Cells were infected in a suspension with HSV-1 at an MOI of 1, then they were incubated for 1 h. The virus suspension was removed and cells were washed with phosphate-buffered saline (PBS). This step was followed by the addition of fresh medium to the cells and they were incubated for 1, 2, 4, 6, 8, or 12 h for the RSII sequencing and for 1, 2, 3, 4, 5, 6, 8, 12, 18, or 24 h for the Sequel and MinION runs.

### RNA purification

#### Total RNA extraction

Total RNA isolation was carried out by using the NucleoSpin® RNA kit (Macherey-Nagel) following the kit’s recommendations and our previously published methods^12^. In sum, the viral infected cells were lysed in a lysis puffer (containing chaotropic ions which inhibit ribonucleases, supplied by the kit). DNase I (provided by the kit) treatment was carried out during the purification. The RNA samples, which were bound to a silica membrane, were eluted in RNase-free water. The potential residual DNA contamination was eliminated by applying additional DNase treatment; the Ambion® TURBO DNA-free^TM^ Kit was used (Thermo Fisher Scientific). The final concentrations of the RNA samples were measured by Qubit® 2.0 Fluorometer using Qubit RNA BR Assay Kit (Life Technologies) and then they were stored at −80 °C until further use. The RNA samples taken from each time points were mixed for library preparation and sequencing.

#### Ribosomal RNA depletion

For the random hexamer-primed cDNA sequencing, the RNA samples were handled with the ribodepletion kit (Epicentre Ribo-Zero^TM^ Magnetic Kit H/M/R, Epicentre/Illumina), to remove the ribosomal (r)RNAs.

#### Isolation of polyadenylated RNA

For the sequencing of the polyadenylated [polyA(+)] fraction of the samples, RNAs were purified by using the Qiagen Oligotex mRNA Mini Kit, following to the “Spin Columns” protocol of the kit.

The final concentrations of the rRNA depleted and the PolyA(+) RNA samples were determined through use of the Qubit RNA HS Assay Kit (Life Technologies).

### Preparation of cDNAs and sequencing libraries

#### PacBio SMRTbell library preparation

Full-length cDNAs were prepared according to the PacBio Isoform Sequencing (Iso-Seq) protocol by using the Clontech SMARTer PCR cDNA Synthesis Kit. We applied the no Size Selection protocol for the study of short RNAs, while we carried out size selection by using SageELF^TM^ and BluePippin^TM^ Size-Selection Systems (Sage Science) for the isolation of long transcripts. Reverse transcription (RT) reactions were primed with oligo(d)T (supplied by the Clontech Kit) or random hexamer primer (custom designed, ordered from IDT DNA). Samples were amplified by PCR using KAPA HiFi Enzyme (Kapa Biosystems), according to the above mentioned PacBio protocol. In short, primary denaturation was done at 95 °C for 2 min. This step was followed by 16 cycles for PA-sequencing, as well as 20 or 30 cycles for random hexamer-primed samples (the optimal cycle was determined in the optimization step) at 98 °C for 20 s (denaturation), 65 °C for 15 s (annealing) 72 °C for 4 min (extension). 72 °C was used for the final extension, it was set to 5 min. (n: 18 cycles was set to the No size-selection protocol, and the same PCR setting was applied for size-selected samples.

PCR products were mixed together and then size selected with the SageELF^TM^ System following the PacBio’s protocol. Size-selected cDNAs were amplified with KAPA enzyme using the above mentioned conditions. The cDNAs with a size over 5 kb was size-selected again, with the BluePippin^TM^ System to remove the short samples. Five-hundred ng of each non-size-selected sample was used for the generation of the SMRTbell templates, with the PacBio DNA Template Prep Kit 1.0. The quantity of the size-selected cDNAs used in the SMRTbell template preparation reaction was based on the following protocols: Procedure & Checklist – Isoform Sequencing (Iso-Seq^TM^) using the Clontech SMARTer PCR cDNA Synthesis Kit and SageELF^TM^ Size Selection System & BluePippin^TM^ Size-Selection Systems. The DNA/Polymerase Binding Kit P6 and v2 primers were used to generate SMRTbell library-DNA polymerase complexes. The ready complexes were bound to MagBeads using the PacBio MagBead Binding Kit.

The volume of the sequencing primer for the annealing, and the polymerase (P5 or P6) for the binding was determined using the PacBio Calculator version 2.3.1.1. by adding the concentrations and the average insert sizes of SMRTbell templates.

The polymerase-template complexes were bound to MagBeads, loaded onto SMRT Cells and sequenced on the RSII instrument.

The Binding Calculator (PacBio) was used to calculate the amount of samples to be used for sequencing, following MagBead one-cell per well (OCPW) method. The P6v2 binding kit was used, the on-plate concentration was set to 0.05 nM. The insert sizes were set based on the applied size-selections: 1000, 2500 and 6000 bp sizes were chosen.

Briefly, the sequencing primer was diluted in PacBio Elution Buffer (EB) to 150 nM. The annealing step was carried out using 1 μl template (cc: ~20 ng/μl), the diluted sequencing primer and primer buffer (10×). The concentration of this reaction mixture was 0.8333 nM. Annealing step was set to 20 °C for 30 min. The DNA polymerase enzyme was diluted to a final concentration of 50 nM in PacBio Binding Buffer (BB) v2, and then it was bound to the annealed sample followed by the addition of DTT, dNTP and BB. The sample complex (final concentration was set to 0.5 nM) was incubated at 30 °C for 4 h. The complex (0.5 μl) was added to 18.5 μl MagBead BB (final concentration was set to 0.0125 nM). The detailed protocol of the MagBeads preparation is as follows: 73.9 μl MagBeads were washed with MagBead Wash Buffer, then it was replaced by MagBead BB (73.9 μl). The washed MagBead was used to bound to the sample-complex. Nineteen μl from the complex was added to the washed MagBeads and then they were incubated in a HulaMixer (Life Technologies) at 4 °C for 30 min. After this binding step, the sample was purified with BB (19 μl), then with 19 μl WB. The final elution was in 19 μl BB. The MagBead-bound sample complexes were loaded for sequencing.

The MagBead One Cell Per Well protocol was applied for the sequencing. Sequencing runs were performed by using the PacBio RS II sequencer or Sequel platform. Size-selected and no size-selected samples were run on RSII, while the Sequel platform was applied for the no size-selected samples. The DNA Sequencing Reagent 4.0 was used for the reactions. Two hundred and forty min or 360 min movie lengths were set for the RSII runs, while 600 min was applied for the Sequel sequencing (one movie was recorded for each SMRT Cell).

### Direct RNA sequencing

To avoid the potential false-priming effect of PCR reactions, an amplification free method was used for the analysis of HSV-1 transcripts. For this, the ONT’s Direct RNA sequencing (DRS) protocol (Version: DRS_9026_v1_revM_15Dec2016) was utilized. A total RNA sample containing an equal amount from the 10 time points (1, 2, 3, 4, 5, 6, 8, 12, 18, 24 h pi) was used to isolate the polyadenylated RNA fraction. This sample (115 ng) was then used as template for the RT, it was added to the RT adapter (supplied by the DRS kit) (Table 7). T4 DNA ligase (2 M U/ml; New England BioLabs) was also added to the reaction. The reaction mixture was incubated at room temperature for 10 min. SuperScript (SS)III RT enzyme (Life Technologies) was added to the RNA-cDNA hybrid, and the generation of the first-strand cDNA was carried out according to the ONT DRS kit’s recommendations: the reaction was done at 50 °C for 50 min, then the SSIII was inactivated at 70 °C (10 min). The RNase OUT (40 U/μl; Life Technologies) recombinant RNase inhibitor–treated Agencourt AMPure XP Magnetic Beads (Beckman Coulter) was used to purify the sample (2U RNase OUT per 1 μl bead), then it was eluted in 20 μl Nuclease-Free Water (Ambion/Thermo Fisher Scientific). T4 DNA ligase and NEBNext Quick Ligation Reaction Buffer (New England BiceoLabs) were used to ligate the purified sample to the RMX adapter. The ligation was done at room temperature. The incubation time was 10 min. The adapter-ligated sample was purified with the RNase OUT-handled XP beads using Wash Buffer (DRS Kit), and then eluted in 21 μl Elution Buffer (DRS Kit). The quantification of the ready libraries was performed using the Qubit 2.0 Fluorometer as well as Qubit dsDNA HS Assay Kit (both from Life Technologies). The R9.4 SpotON Flow Cell was used for MinION sequencing.

### Oxford Nanopore 1D cDNA sequencing

Samples were sequenced on a MinION device from the ONT according to the ONT 1D Strand switching cDNA by ligation protocol (Version: SSE_9011_v108_revS_18Oct2016). The sequencing libraries were generated by using the ONT Ligation Sequencing Kit 1D (SQK-LSK108). The PolyA(+)-selected RNA fraction or the rRNA-depleted sample was used for cDNA production. Identical quantity of RNA samples from each of the applied time points were mixed together.

Thirty-one ng from the Poly(A^+^)-selected, or 50 ng from the rRNA-depleted sample was used as template for the RT reactions. An anchored oligo(d)T primer [(VN)T20; ordered from Bio Basic, Canada, (Table 7)] or a modified random-primer (Table 7) was used for priming the reactions. The RNA-primer mixture was incubated at 65 °C for 5 min. After this short step, a strand-switching oligo [containing three O-methyl-guanine RNA bases (PCR_Sw_mod_3G; Bio Basic, Canada)], buffer and DTT [both are derived from the SuperScript IV Reverse Transcriptase kit (Life Technologies)] were added to the reactions. The samples were handled with a recombinant RNase inhibitor (RNase OUT^TM^, Life Technologies); the incubation was carried out at 42 °C 2 min.

The SuperScript IV Reverse Transcriptase enzyme (200 unit) was added to the sample. The RT reaction was carried out in a Veriti Cycler (Applied Biosystems) at 50 °C for 10 min. It was followed by the strand-switching step at 42 °C for 10 min. Samples were heated to 80 °C (10 min) to inactivate the enzymes. Samples were amplified by PCR with KAPA HiFi DNA Polymerase (Kapa Biosystems) and Ligation Sequencing Kit Primer Mix (1D Kit). Five μl from the cDNAs were used as template in each of the PCR reactions. The Veriti PCR machine was set as follows: initial denaturation 95 °C, 30 sec (1 cycle); denaturation 95 °C, 15 sec (15 cycles); annealing 62 °C 15 sec (15 cycles); extension 65 °C 4 min (15 cycles); final extension step 65 °C, 10 min. NEBNext End repair/dA-tailing Module (New England Biolabs) was utilized for repairing the DNA ends, while the NEB Blunt/TA Ligase Master Mix (New England Biolabs) was used for ligating the adapters (1D kit). The Beckman Coulter Agencourt AMPure XP beads were used to purify the DNA after each of the enzymatic steps. The concentration of the samples was measured by using the Qubit Fluorometer 2.0 (Life Technologies) and the Qubit (ds)DNA HS Assay Kit (Life Technologies). The libraries were sequenced on the ONT R9.4 SpotON Flow Cells.

### MinION cDNA sequencing on Cap-selected samples

To generate full-length cDNA from capped and polyadenylated RNAs, the “all-in-one” protocol from the Lexogen was applied using the TeloPrime Full-Length cDNA Amplification Kit. A 2.2 μg mixture from the different total RNAs (1, 2, 3, 4, 5, 6, 8, 12, 18 and 24 h pi) was used for the RT. For this, the sample was mixed with RT buffer and a specific primer (both are the part of the kit, Table 7). The reaction started at a 30 sec incubation at 70 °C, then it was followed by a 1 min step at 37 °C. At this point, the reaction was kept at 37 °C. The reverse transcriptase and the additional reagents (derived from the kit) were mixed with the sample and then the incubation was containing at 37 °C for 2 min. The next step of the RT reaction was carried out at 46 °C for 50 min. Sample was purified by using the kit’s Silica columns. The double-strand (ds) specific ligase enzyme (Lexogen kit) was used to join the adapter to the cDNA, the reaction was performed at 25 °C, overnight, then the sample was purified using the silica membranes of the Lexogen kit. The dscDNAs were generated by using the Enzyme Mix and the Second-Strand Mix (Lexogen kit). The cDNA production was performed in a Veriti cycler. The following protocol was applied: 98 °C for 90 sec, 62 °C for 60 sec, 72 °C for 5 min (16 cycles), hold at 25 °C. The sample concentration was detected by using Qubit 2.0 and Qubit dsDNA HS quantitation assay (Life Technologies). The specificity of the obtained product was checked by using real-time PCR. The Rotor-Gene Q qPCR machine (Qiagen), a gene specific primer (*us9*, 10 μM each, ordered from IDT DNA, Table 8), and the ABsolute qPCR SYBR Green Mix (Thermo Fisher Scientific) was applied. The preliminary denaturation was carried out at 94 °C for 15 min, then 35 cycles of 94 °C for 25 sec, 60 °C 25 sec and 72 °C 6 sec was applied.

The cDNAs from polyadenylated-capped RNA samples were used for library preparation for ONT sequencing. The 1D Strand switching cDNA by ligation method was used. After the end-repair, the samples were ligated to the 1D adapters. Finally, they were measured on the ONT R9.4 SpotON Flow Cells.

### Read processing

The SMRT Analysis v2.3.0 (PacBio RSII), the SMRT Link (PacBio Sequel) and the Albacore v2.0.1 (ONT MinION) software packages were used for base calling (Figure 1). The reads were mapped by using GMAP and the following setting was applied: Minimum Full Passes = 1, Minimum Predicted Accuracy = 90, Minimum Length of Reads of Insert = 1, Maximum Length of Reads of Insert = No Limit. These consensus reads were mapped using GMAP^17^, with the following settings: gmap -d Genome.fa --nofails -f samse File.fastq > Mapped_file.sam. The quality information was acquired by using custom made routines^18^.

The Porechop v.0.2.3 software was used to determine the orientation of the sequencing reads. For this, a modified adapter.py file was used, where we added the various, library-specific adapter sequences (Table 6). The first mapped nucleotide downstream the “END” adapter was labelled the 5′ end of a read, while the last mapping nucleotide upstream of the “START” (polyA tail: A_20_ or 3′ adapters) was designated the 3′ end of the read. Reads lacking an adapter on both ends, or with 5′ or 3′ adapters on both ends, can be discarded from further *in silico* analysis.

### Code Availability

1.SMRT Analysis v2.3.0: https://s3.amazonaws.com/files.pacb.com/software/smrtanalysis/2.3.0/smrtanalysis-patch_2.3.0.140936.p5.run

2.SMRT Link v5.1.0: https://downloads.pacbcloud.com/public/software/installers/smrtlink_5.1.0.26412.zip

3.Albacore v2.0.1: https://github.com/Albacore/albacore

4.GMAP: http://research-pub.gene.com/gmap/ (version 2015-12-31)

5.Custom routines have been archived on Github (https://doi.org/10.5281/zenodo.1034511).

6.Porechop v.0.2.3: https://github.com/rrwick/Porechop

## Data Records

Data from MinION and Sequel sequencing have been uploaded to the European Nucleotide Archive (Data Citation 1) - contains BAM files. The RSII raw sequencing files, processed data files as well as metadata have been submitted to the Gene Expression Omnibus repository (Data Citation 2). All reads were mapped to the X14112 genome build. The provided sequencing data can be used without restrictions.

## Technical Validation

The quantity of the purified total RNAs, the polyA-selected RNAs, the ribodepleted RNA samples, as well as the cDNA samples and the final sequencing libraries were detected by Qubit 2.0 (Life Technologies) fluorometer using the Qubit RNA Broad-Range, High Sensitivity RNA and High Sensitivity dsDNA Assay Kits.

## Usage Notes

Our provided dataset was primarily generated to analyse the potential splice variants, as well as transcriptional start and stop site variations, the isoforms and the complexity of HSV-1 transcriptome. The provided raw RSII data files can be utilized to develop novel base calling algorithms or read processing tools, as well as to improve the currently existing bioinformatics software. The uploaded BAM files contain reads already aligned to the X14112 HSV-1 reference genome using GMAP v2017-04-24^17^.

These mapped reads can be further analysed by using different long-read aligners (e.g. NGMLR: https://github.com/philres/ngmlr; GraphMap: https://github.com/isovic/graphmap, etc.), or bioinformatics tools (e.g. samtools^19^ and bedtools^20^). These data can be visualized by utilizing different programs such as the Geneious^21^, IGV^22^, or Artemis^23^. The files contain terminal poly(A) sequences as well as the 5′and 3′ adapter sequences, which can be utilized to determine the orientations of the reads. Novel long-read sequencing pipelines e.g.: SQANTI^24^ can be tested using this dataset.

## Additional information

**How to cite this article**: Boldogkői, Z. *et al*. Transcriptomic study of Herpes simplex virus type-1 using full-length sequencing techniques. *Sci. Data*. 5:180266 doi: 10.1038/sdata.2018.266 (2018).

**Publisher’s note**: Springer Nature remains neutral with regard to jurisdictional claims in published maps and institutional affiliations.

## Supplementary Material



## Figures and Tables

**Table 1:** Summary of the obtained read counts aligned to the HSV-1 reference genome.

Sample	Mapped read count
RSII PolyA size selected: 0.8–2 kb	5689
RSII PolyA size selected: 2–3 kb	3669
RSII PolyA size selected: 3–5 kb	12431
RSII PolyA size selected: 5 kb^+^	7010
RSII PolyA non size selected	10796
RSII random-primed	516
Sequel	78362
MinION PolyA and Cap selected cDNA seq	36386
MInION PolyA cDNA seq	67475
MinION random-primed cDNA seq	60687
MInION direct RNA seq	16997

**Table 2:** Summary table of the obtained read lengths from PacBio RSII and Sequel, as well as from ONT MinION sequencing.

Sample	Median read lengths	Average read lengths ±SD	Median aligned read lengths	Average aligned read lengths±SD
RSII PolyA size selected: 0.8-2kb	1374	1385 ± 341,29	1286	1283 ± 316,64
RSII PolyA size selected: 2-3kb	1442	1543 ± 538,90	1346	1442 ± 503,44
RSII PolyA size selected: 3-5kb	1355	1333 ± 503,22	1241	1216 ± 462,89
RSII PolyA size selected: 5kb^+^	1724	1815 ± 794,21	1605	1588 ± 739,83
RSII PolyA non size selected	1434	1536 ± 607,55	1338	1372 ± 488,48
RSII random-primed	903	1000 ± 459,25	830	925 ± 461,45
Sequel	1823	2104 ± 814,41	1723	1952 ± 768,24
MinION PolyA and Cap selected cDNA seq	999	1067 ± 598,68	620	709 ± 355,75
MInION PolyA cDNA seq	1313	1466 ± 844,69	1000	1046 ± 679,91
MinION random-primed cDNA seq	801	910 ± 521,04	717	822 ± 491,69
MInION direct RNA seq	1041	1111 ± 673,45	904	948 ± 565,86

**Table 3:** Summary table of the various wet lab methods utilized in this project.

Sample No	Sample	Sample time points (h pi)	RT priming	Cap-selection	Amplification	Size selection	Library prep	Platform
1	Mixed	1, 2, 4, 6, 8, 12	Oligo(d)T	no	yes	Bluepippin 0.8-2kb	PacBio Isoform seq	RSII
2	Mixed	1, 2, 4, 6, 8, 12	Oligo(d)T	no	yes	Bluepippin 2-3kb	PacBio Isoform seq	RSII
3	Mixed	1, 2, 4, 6, 8, 12	Oligo(d)T	no	yes	Bluepippin 3-5kb	PacBio Isoform seq	RSII
4	Mixed	1, 2, 4, 6, 8, 12	Oligo(d)T	no	yes	Bluepippin 5kb+	PacBio Isoform seq	RSII
5	Mixed	1, 2, 4, 6, 8, 12	Oligo(d)T	no	yes	no	PacBio Isoform seq	RSII
6	Mixed	1, 2, 4, 6, 8, 12	Random	no	yes	no	PacBio Isoform seq	RSII
7	Mixed	1, 2, 3, 4, 5, 6, 8, 12, 18, 24	Oligo(d)T	no	yes	no	PacBio Isoform seq	Sequel
8	Mixed	1, 2, 3, 4, 5, 6, 8, 12, 18, 24	Oligo(d)T	yes	yes	no	ONT cDNA	MinION
9	Mixed	1, 2, 3, 4, 5, 6, 8, 12, 18, 24	Oligo(d)T	no	yes	no	ONT cDNA	MinION
10	Mixed	1, 2, 3, 4, 5, 6, 8, 12, 18, 24	Random	no	yes	no	ONT cDNA	MinION
11	Mixed	1, 2, 3, 4, 5, 6, 8, 12, 18, 24	Oligo(d)T	no	no	no	ONT Direct RNA	MinION

**Table 4:** Summary of the read qualities gained from the utilized long-read sequencing approaches.

Sample	Percentage of deletions (%) ± SD	Percentage of insertions (%) ± SD	Percentage of mismatches (%) ± SD	Coverage
RSII PolyA size selected: 0.8-2kb	0,84 ± 1,25	0,35 ± 0,88	0,76 ± 0,49	47.70
RSII PolyA size selected: 2-3kb	0,79 ± 1,22	0,41 ± 0,91	0,74 ± 0,46	34.40
RSII PolyA size selected: 3-5kb	0,75 ± 1,25	0,38 ± 1,01	0,77 ± 0,56	98.24
RSII PolyA size selected: 5kb^+^	0,87 ± 1,67	0,94 ± 3,43	1,52 ± 3,19	66.93
RSII PolyA non size selected	0,90 ± 1,27	0,61 ± 1,18	0,72 ± 0,50	95.43
RSII random-primed	0,92 ± 1,68	0,23 ± 0,78	0,71 ± 0,49	3.01
Sequel	0,41 ± 0,64	1,18 ± 2,53	0,78 ± 0,61	998.24
MinION PolyA and Cap selected cDNA seq	7,43 ± 2,08	3,09 ± 2,15	8,81 ± 2,44	161.48
MInION PolyA cDNA seq	7,74 ± 2,06	3,61 ± 2,17	8,75 ± 2,47	444.16
MinION random-primed cDNA seq	5,41 ± 2,40	3,75 ± 2,24	6,63 ± 2,23	320.17
MInION direct RNA seq	9,76 ± 2,24	2,72 ± 2,12	8,36 ± 1,98	102.81

**Table 5:** Statistics of the different libraries. This table shows the results of the adapter analysis using the Porechop tool.

Sequencer	Library spec	Orientation	Subsample	Adapters	Subsample read count	Read count	
**RSII**	**0.8-2kb**	**No**	**No adapter**		56	56	5659
			**Adapters at the same end of a read**	PacBio and/or PolyA	2	2	
			**Same adapter at both end of a read**	PacBio and PolyA	42	106	
				PacBio	36		
				PolyA	28		
		**Yes**	**Fw**	PacBio and PolyA	3182	3336	
				PacBio	51		
				PolyA	103		
			**Rev**	PacBio and PolyA	2061	2159	
				PacBio	27		
				PolyA	71		
	**2-3kb**	**No**	**No adapter**		55	55	3633
			**Adapters at the same end of a read**	PacBio and/or PolyA	10	10	
			**Same adapter at both end of a read**	PacBio and PolyA	131	168	
				PacBio	28		
				PolyA	9		
		**Yes**	**Fw**	PacBio and PolyA	1796	1955	
				PacBio	64		
				PolyA	95		
			**Rev**	PacBio and PolyA	1327	1445	
				PacBio	48		
				PolyA	70		
	**3-5kb**	**No**	**No adapter**		553	553	12299
			**Adapters at the same end of a read**	PacBio and/or PolyA	168	168	
			**Same adapter at both end of a read**	PacBio and PolyA	2001	2377	
				PacBio	316		
				PolyA	60		
		**Yes**	**Fw**	PacBio and PolyA	5067	5887	
				PacBio	334		
				PolyA	486		
			**Rev**	PacBio and PolyA	2823	3314	
				PacBio	207		
				PolyA	284		
	**>5kb**	**No**	**No adapter**		95	95	6417
			**Adapters at the same end of a read**	PacBio and/or PolyA	28	28	
			**Same adapter at both end of a read**	PacBio and PolyA	284	466	
				PacBio	117		
				PolyA	65		
		**Yes**	**Fw**	PacBio and PolyA	3096	3487	
				PacBio	122		
				PolyA	269		
			**Rev**	PacBio and PolyA	2058	2341	
				PacBio	54		
				PolyA	229		
	**polyA**	**No**	**No adapter**		355	355	10593
			**Adapters at the same end of a read**	PacBio and/or PolyA	19	19	
			**Same adapter at both end of a read**	PacBio and PolyA	149	553	
				PacBio	206		
				PolyA	198		
		**Yes**	**Fw**	PacBio and PolyA	5435	6277	
				PacBio	334		
				PolyA	508		
			**Rev**	PacBio and PolyA	2906	3389	
				PacBio	176		
				PolyA	307		
	**random**	**No**	**No adapter**		56	56	495
			**Same adapter at both end of a read**	PacBio	157	157	
		**Yes**	**Fw**	PacBio	157	157	
			**Rev**	PacBio	125	125	
**Sequel**		**No**	**No adapter**		58	58	77851
			**Adapters at the same end of a read**	PacBio and/or PolyA	97	97	
			**Same adapter at both end of a read**	PacBio and PolyA	2620	4874	
				PacBio	1706		
				PolyA	548		
		**Yes**	**Fw**	PacBio and PolyA	41895	44238	
				PacBio	1738		
				PolyA	605		
			**Rev**	PacBio and PolyA	26378	28584	
				PacBio	1753		
				PolyA	453		
**MinION**	**polyA**	**No**	**No adapter**		7636	7636	64681
			**Adapters at the same end of a read**	PCR-1, SS, PCR-2, PolyA	97	5362	
				PCR-1, SS, PCR-2	14		
				PCR-1, SS, PolyA	246		
				PCR-1, SS	106		
				PCR-1, PCR-2, PolyA	34		
				PCR-1, PCR-2	5		
				PCR-1, PolyA	23		
				SS, PCR-2, PolyA	2004		
				SS, PCR-2	169		
				SS, PolyA	2167		
				PCR-2, PolyA	497		
			**Same adapter at both end of a read**	PCR-1, SS, PCR-2, PolyA	98	8896	
				PCR-1, SS, PCR-2	12		
				PCR-1, SS, PolyA	275		
				PCR-1, SS	103		
				PCR-1, PCR-2, PolyA	12		
				PCR-1, PolyA	25		
				PCR-1	2		
				SS, PCR-2, PolyA	1032		
				SS, PCR-2	68		
				SS, PolyA	1007		
				SS	255		
				PCR-2, PolyA	3395		
				PCR-2	29		
				PolyA	2583		
		**Yes**	**Fw**	PCR-1, SS, PCR-2, PolyA	284	22391	
				PCR-1, SS, PCR-2	40		
				PCR-1, SS, PolyA	587		
				PCR-1, SS	354		
				PCR-1, PCR-2, PolyA	418		
				PCR-1, PCR-2	82		
				PCR-1, PolyA	906		
				PCR-1	553		
				SS, PCR-2, PolyA	1882		
				SS, PCR-2	238		
				SS, PolyA	1908		
				SS	1634		
				PCR-2, PolyA	5530		
				PCR-2	892		
				PolyA	7083		
			**Rev**	PCR-1, SS, PCR-2, PolyA	207	20396	
				PCR-1, SS, PCR-2	31		
				PCR-1, SS, PolyA	509		
				PCR-1, SS	262		
				PCR-1, PCR-2, PolyA	383		
				PCR-1, PCR-2	61		
				PCR-1, PolyA	942		
				PCR-1	497		
				SS, PCR-2, PolyA	1528		
				SS, PCR-2	149		
				SS, PolyA	1666		
				SS	994		
				PCR-2, PolyA	5330		
				PCR-2	640		
				PolyA	7197		
	**random**	**No**	**No adapter**	27540	27540	59291	
			**Adapters at the same end of a read**	SS, PolyA	10	10	
			**Same adapter at both end of a read**	SS, PolyA	4	2865	
				SS	2861		
		**Yes**	**Fw**	SS, PolyA	17	13242	
				SS	13176		
				PolyA	49		
			**Rev**	SS, PolyA	16	15634	
				SS	15568		
				PolyA	50		
	**Cap**	**No**	**No adapter**		1649	1649	34681
			**Adapters at the same end of a read**	CAP5, CAP3, CAP3-pA, PolyA	307	3346	
				CAP5, CAP3, CAP3-pA	33		
				CAP5, CAP3, PolyA	515		
				CAP5, CAP3	1554		
				CAP5, CAP3-pA, PolyA	27		
				CAP5, CAP3-pA	1		
				CAP5, PolyA	26		
				CAP3, CAP3-pA, PolyA	430		
				CAP3, CAP3-pA	16		
				CAP3, PolyA	431		
				CAP3-pA, PolyA	6		
			**Same adapter at both end of a read**	CAP5, CAP3, CAP3-pA, PolyA	1404	9524	
				CAP5, CAP3, CAP3-pA	1015		
				CAP5, CAP3, PolyA	262		
				CAP5, CAP3	2379		
				CAP5, CAP3-pA, PolyA	22		
				CAP5, CAP3-pA	1		
				CAP5, PolyA	30		
				CAP5	424		
				CAP3, CAP3-pA, PolyA	1622		
				CAP3, CAP3-pA	758		
				CAP3, PolyA	216		
				CAP3	1241		
				CAP3-pA, PolyA	123		
				PolyA	27		
		**Yes**	**Fw**	CAP5, CAP3, CAP3-pA, PolyA	1263	10575	
				CAP5, CAP3, CAP3-pA	977		
				CAP5, CAP3, PolyA	338		
				CAP5, CAP3	2285		
				CAP5, CAP3-pA, PolyA	228		
				CAP5, CAP3-pA	22		
				CAP5, PolyA	544		
				CAP5	1544		
				CAP3, CAP3-pA, PolyA	683		
				CAP3, CAP3-pA	505		
				CAP3, PolyA	170		
				CAP3	1529		
				CAP3-pA, PolyA	180		
				CAP3-pA	17		
				PolyA	290		
			**Rev**	CAP5, CAP3, CAP3-pA, PolyA	1312	9587	
				CAP5, CAP3, CAP3-pA	703		
				CAP5, CAP3, PolyA	297		
				CAP5, CAP3	1452		
				CAP5, CAP3-pA, PolyA	235		
				CAP5, CAP3-pA	16		
				CAP5, PolyA	505		
				CAP5	1411		
				CAP3, CAP3-pA, PolyA	905		
				CAP3, CAP3-pA	513		
				CAP3, PolyA	165		
				CAP3	1474		
				CAP3-pA, PolyA	248		
				CAP3-pA	8		
				PolyA	343		
	**dRNA**	No	**No adapter**		11596	11596	16516
			**Adapters at the same end of a read**	SS,PolyA,	36	36	
			**Same adapter at both end of a read**	SS,PolyA,	9	375	
				SS,	366		
		Yes	**Fw**	SS,PolyA,	33	3015	
				SS,	2717		
				PolyA,	265		
			**Rev**	SS,PolyA,	41	1494	
				SS,	1195		
				PolyA,	258		
Using this script, we were able to identify the adapter sequences (Table 6) at the 5’ and 3’ ends of the reads, and thus, determine their orientations. We were able to define the orientation of more than 75% of the PacBio reads in every library, however we obtained poorer results using the MinION (due to the lower read quality): 66% of the reads from the 1D polyA libraries were identified as forward or reverse-oriented reads, while these values were only 58% and 49% from the Cap-seq and random libraries, respectively.							

**Table 6:** This table shows the adapter sequences of the various library-preparation methods, which were used to determine the orientation of transcripts.

	START	END
MinION cDNA	TTACGGCCGGG	CCCGGCCGTAA
CAP 5’	CTCACTATAG	CTATAGTGAGT
CAP 3’	TCTCAGGCG	CGCCTGAGA
CAP 3’ PolyA	TCTCAGGCGTTTTTTTTTTTTTTTTTT	AAAAAAAAAAAAAAAAAACGCCTGAGA
PolyA	TTTTTTTTTTTTTTTTTTTT	AAAAAAAAAAAAAAAAAAAA
PacBio	AGAGTACATGGG	CCCATGTACTCT
PCR tail 1	TTAACCTTTCTGTTGGTGCTGATATTGC	GCAATATCAGCACCAACAGAAAGGTTAA
PCR tail 2	TTAACCTACTTGCCTGTCGCTCTATCTTC	GAAGATAGAGCGACAGGCAAGTAGGTTAA

**Table 7:** The list of reverse transcription primers used in this study.

Sequencing method	Name, availability	Catalog #	Sequence (5′ -> 3′)
PacBio amplified PolyA	3' SMART CDS primer II A - SMARTer PCR cDNA Synthesis Kit (Clontech)	634925 & 634926	AAGCAGTGGTATCAACGCAGAGTAC(T)_30_VN
PacBio amplified Random	Custome made (IDT DNA)	—	AAGCAGTGGTATCAACGCAGAGTACNNNNNN (G: 37%; C: 37%; A: 13%; T: 13%)
MinION CAP selected	TeloPrime Full-Length cDNA Amplification Kit (Lexogen)	013.08 & 013.24	TCTCAGGCGTTTTTTTTTTTTTTTTTT
MinION cDNA	Poly(T)-containing anchored primer [(VN)T20 - ONT recommended, custom made (Bio Basic)	—	5phos/ACTTGCCTGTCGCTCTATCTTC(T)_20_VN
MinION Random	Custome made (IDT DNA)	—	5Phos/ACTTGCCTGTCGCTCTATCTTCNNNNNN
MinION RNA	RT adapter - Direct RNA Sequencing Kit (Oxford Nanopore Technologies)	SQK-RNA001	GAGGCGAGCGGTCAATTTTCCTAAGAGCAAGAAGAAGCCTTTTTTTTTT

**Table 8:** The sequence of the primer-pair used for the detection of *us9* gene of HSV-1 by qPCR.

Primer	Sequence
Forward	GGCTGCTCCGCTAAAAGAC
Reverse	AGTTAAAGGCTGGGTGCAAA
